# Effects of Dietary Ferulic Acid on the Intestinal Microbiota and the Associated Changes on the Growth Performance, Serum Cytokine Profile, and Intestinal Morphology in Ducks

**DOI:** 10.3389/fmicb.2021.698213

**Published:** 2021-07-13

**Authors:** Yang Liu, Qian Lin, Xuan Huang, Guitao Jiang, Chuang Li, Xu Zhang, Shengli Liu, Lingyun He, Yali Liu, Qiuzhong Dai, Xingguo Huang

**Affiliations:** ^1^College of Animal Science and Technology, Hunan Agriculture University, Changsha, China; ^2^Hunan Institute of Animal and Veterinary Science, Changsha, China; ^3^Institute of Bast Fiber Crops, Chinese Academy of Agricultural Sciences, Changsha, China; ^4^Shandong Lonct Enzymes Co., Ltd., Linyi, China; ^5^Animal Husbandry and Fisheries Affairs Center, Huaihua, China; ^6^Hunan Perfly Biotech Co., Ltd., Changsha, China

**Keywords:** gut microbiota, ferulic acid, growth performance, serum cytokine, intestinal morphology, duck

## Abstract

The present study investigated the effects of ferulic acid (FA) on the growth performance, serum cytokine profile, intestinal morphology, and intestinal microbiota in ducks at the growing stage. 300 female *Linwu* ducks at 28 days of age with similar body weights were randomly divided into five groups. Each group contained six replicates of 10 birds. The dietary treatments were corn-soybean-based diet supplemented with FA at the concentrations of 0 (control), 100, 200, 400, and 800 mg/kg diet. The results demonstrated that dietary FA at the levels of 200, 400, and 800 mg/kg increased the average daily gain (*P* = 0.01), 400 and 800 mg/kg FA increased the final body weight (*P* = 0.02), 100, 200, and 800 mg/kg FA increased the serum glutathione (*P* = 0.01), and 100, 400, and 800 mg/kg FA increased the glutathione peroxidase activities in birds (*P* < 0.01). Additionally, 200, 400, and 800 mg/kg dietary FA lowered the serum levels of interleukin-2 (*P* = 0.02) and interleukin-6 (*P* = 0.04). Moreover, the morphometric study of the intestines indicated that 400 mg/kg FA decreased the crypt depth in jejunum (*P* = 0.01) and caecum (*P* = 0.04), and increased the ratio of villus height to crypt depth in jejunum (*P* = 0.02). Significant linear and/or quadratic relationships were found between FA concentration and the measured parameters. 16S rRNA sequencing revealed that dietary FA increased the populations of genera *Faecalibacterium*, *Paludicola*, RF39, and *Faecalicoccus* in the cecum (*P* < 0.05), whereas decreased the populations of *Anaerofilum* and UCG-002 (*P* < 0.05). The Spearman correlation analysis indicated that phylum *Proteobacteria* were negatively, but order *Oscillospirales*, and family *Ruminococcaceae* were positively related to the parameters of the growth performance. Phylum *Bacteroidetes*, class *Negativicutes* and family *Rikenellaceae* were negatively associated with the parameters of the antioxidative capability. And phylum *Cyanobacteria*, *Elusimicrobia*, and *Bacteroidetes*, class *Bacilli*, family *Rikenellaceae*, and genus *Prevotella* were positively associated with the parameters of the immunological capability. Thus, it was concluded that the supplementations of 400 mg/kg FA in diet was able to improve the growth performance, antioxidative and immunological capabilities, intestinal morphology, and modulated the gut microbial construction of *Linwu* ducks at the growing stage.

## Introduction

The intensive rearing system for poultry was widespread throughout the world. Better quantity and quality of poultry products were expected by breeding birds in indoor system with advanced technologies and management skills (Robins and Phillips, 2011). The intensive rearing system satisfied the increasing demands for the poultry product, but also brought many problems to the welfare of the birds, including the physiological and physical stresses (Lolli et al., 2010; Averós and Estevez, 2018), pathogenic infections (Zhu, 2011), and disturbance in the intestinal microbial construction (Wang S. et al., 2018). These problems might break the redox homeostasis and provoked immune response (Rehman et al., 2017; Lauridsen, 2018), leading to high mortality and lower productive efficiency (Averós and Estevez, 2018). In the past, antibiotic additives in poultry feed were commonly used in the intensive rearing system because they could effectively attenuate the negative influence of stress, and enhance the growth performance of birds (Castanon, 2007). However, the abuse of antibiotics led to the development of bacterial antibiotic-resistance and created huge pressure on the medication of bacterial infections in human and animal (Hayes et al., 2004). Starting from 2006, legislative bans on antibiotics in animal feed were issued worldwide gradually.

On this background, phytochemicals with characteristics of safety, multiple bioactivities and enhancement to animal growth became ideal alternations to the antibiotic additives in animal feed (Valenzuela-Grijalva et al., 2017). Ferulic acid (FA) is an active phenolic acid widely existing in the cell walls of plants, and it is the main active substance of Chinese herbs Chuangxiong (*Ligusticum chuanxiong*) and Danggui (*Angelica sinensis*) (Ou and Kwok, 2004). Previous researchers stated that FA possessed multiple bioactivities, such as anti-bacteria (Daglia, 2012), antioxidant (Mahmoud et al., 2020), anti-inflammatory (Lampiasi and Montana, 2016), anti-obesity (Wang et al., 2015), anti-tumor (Jinhua et al., 2018), and regulation of blood circulation (Zhou et al., 2017). Currently, FA was widely used in industries such as food preservation (Ou et al., 2005), cosmetics production (Gupta et al., 2020), and medication (Wang W. M. et al., 2018). A few studies demonstrated the antioxidative and growth promotional functions of FA and its derivatives in livestock, as supplement in the feed (Li et al., 2015; González-Ríos et al., 2016; Wang et al., 2019), but none referred to ducks. In the present study, we attempted to understand the effects of FA on the growth performance, serum cytokine profiles, intestine morphology, and intestinal microbiota composition in ducks.

## Materials and Methods

The experimental procedures of this study were approved by Hunan Agricultural University Institutional Animal Care and Use Committee.

### Experimental Design and Diets

*Linwu* duck is an important indigenous breed in South China, with the characteristics of fast growth, high feed efficiency, and unique meat flavor and texture. The market age for *Linwu* duck is 70 days and the average body weight at that time is about 1,750 g per duck. From the age of 28–54 days was the maximum growing stage for *Linwu* ducks, when they suffered the most by the immune and oxidative stresses. Therefore, *Linwu* ducks at 28 days of age were chosen for this study. A total of 300 female ducks were obtained from Hunan Shunhua Duck Industrial Development Company (Linwu, China) and housed in plastic plain netting cages with the dimension of 1.8 m × 1.2 m × 2 m (10 ducks/cage). Ducks were accessed to the water and feed freely. Birds were randomly divided into five groups with six replicates per group. And each replicate had 10 birds. The whole experiment period was 29 days.

Birds in group 1 were fed the basal diets, and in group 2–5 were fed basal diets supplemented with 100, 200, 400, and 800 mg/kg FA, respectively. The basal diet, which meets the nutritional requirements for growing ducks (National Research Council, 1994), was given in Table 1. FA (≥99%) was purchased from Shanghai Rhawn Chemical Technology Co., Ltd. (Shanghai, China).

**TABLE 1:** Ingredients and nutrient composition of the basal diet (dry matter basis, %).

**Ingredients**		**Nutrient levels^1^**	
Corn	50.68	Metabolic energy, MJ/kg	11.30
Soybean meal	24.50	Dry matter (DM)	87.3
Flour	10.00	Crude protein (CP)	17
Wheat middlings	7.00	Calcium (Ca)	0.90
CaHPO_4_	1.30	Total phosphorus (TP)	0.56
Salt	0.30	Available phosphorus (AP)	0.35
*L*-Lysine H_2_SO_4_	0.27	Salt	0.33
*DL*-Methionine	0.12	Lysine	0.9
Limestone	1.20	Methionine	0.4
Bentonite	3.63	Methionine and cystine	0.789
Premix^2^	1.00	Isoleucinese	0.732
		Threonine	0.6
		Tryptophane	0.264

*^1^The nutrient levels were calculated values. ^2^The premix provided the following nutrients per kg diet: vitamin A 12,000 IU; vitamin D_3_ 2,500 IU; vitamin E 20 mg; vitamin K_3_ 3 mg; vitamin B_1_ 3 mg; vitamin B2 8 mg; vitamin B6 7 mg; vitamin B12 0.03 mg; *D*-pantothenic acid 20 mg, nicotinic acid 50 mg, biotin 0.1 mg, folic acid 1.5 mg, Cu (as copper sulfate) 9 mg, Zn (as zinc sulfate) 110 mg, Fe (as ferrous sulfate) 100 mg, Mn (as manganese sulfate) 100 mg, Se (as sodium selenite) 0.16 mg, and I (as potassium iodide) 0.6 mg.*

### Growth Performance

The birds were fasted for 12 h before weighting and sampling. On 29th day of the experiment, birds were weighted, and the total feed consumptions were summed up by cages (replicates). Average daily weight gain (ADG), average daily feed intake (ADFI) and ratio of feed to gain (F/G) were calculated for the whole experimental period.

### Sample Collections

After weighting, one bird from each cage (6 birds per group) was randomly selected for sampling. Bloods were collected from the wing vein with vacuum blood collection tubes, and centrifuged at 3,000 × *g* for 10 min to obtain the serums. The birds were then sacrificed by bleeding in jugular vein and dissected for the duodenum, jejunal, ileal, and cecal tissue samples. Chyme in the cecum of each sampled duck were collected separately into 2 mL EP tubes, froze immediately in liquid nitrogen and stored at −80°C until analysis.

### Serum Antioxidant and Inflammatory Biomarkers

The serum levels of antioxidant biomarkers including reduced glutathione (GSH), malonaldehyde (MDA), activities of superoxide dismutase (SOD) and glutathione peroxidase (GSH-Px), as well as the inflammatory biomarkers, such as immunoglobulin G (IgG), interlukin-1β (IL-1β), interlukin-6 (IL-6), and interleukin-2 (IL-2) were determined by the commercial assay kits (Nanjing Jiancheng Bioengineering Institute, Nanjing, China) with an automated fluorescence instrument (Multiskan^TM^ SkyHigh, Thermo Fisher Scientific, Waltham, MA, United States).

### Intestinal Morphology

The intestinal sections, including duodenum, jejunum, ileum, and cecum, were removed and emptied. 2 cm sections of intestinal tissues were collected and embedded in paraffin. A microtome (RM-2235, Leica microsystems AG, Hessen, Germany) was used to make 5 μm slices of the tissue samples, which were subsequently stained with hematoxylin and eosin. The slides were observed under a microscope (Olympus Van-Ox S, Opelco, Washington, DC, United States) and the proper microscopic fields were selected for the following diagnoses. Five readings of the villus height, crypt depth, intestinal wall thickness and mucosal thickness from each slide were determined by an image analysis system (Image-Pro, Media Cybernetics, Inc., Silver Springs, MD, United States). The ratios of villus height to crypt depth (VCR) were calculated as well.

### Gut Microbiota Composition by 16S rRNA Gene Sequencing

Microbial community genomic DNA was extracted from cecum digesta samples using the E.Z.N.A. soil DNA kits (Omega Bio-tech, Norcross, GA, United States) according to the manufacturer’s instructions. The V3-V4 regions of the bacterial 16S rRNA gene were amplified using primer pairs 338F (5′-ACTCCTACGGGAGGCAGCAG-3′) and 806R (5′-GGACTACHVGGGTWTCTAAT-3′), and later purified using AxyPrep DNA Gel Extraction Kits (Axygen Bioscience, Union City, CA, United States). The purified amplicons were paired-end sequenced on an Illumina MiSeq PE300 platform (Illumina, San Diego, CA, United States). The raw reads in this study were uploaded to the National Center of Biotechnology Information (NCBI) Sequence Read Archive (SRA) database under accession number PRJNA723283.

Reads with length over 300 bp were kept for the following analysis. QIIME (version 1.17) software was used to filter the obtained sequences from the samples and the high-quality sequences were clustered into operational taxonomic units (OTUs) with a cut off of 97% similarity using UPARSE (version 7.1). Each OTU represented sequence was analyzed by Ramer–Douglas–Peucker (RDP) Classifier version 2.2 against the 16S rRND database.

The alpha and beta diversities of the cecal microbiota were estimated at genera level. Principal coordinate analysis (PCA) with analysis of similarities (ANOSIM) was carried out to determine the beta diversity of the bacteria. Statistical comparison of the relative abundance of the cecal microbiota was performed using one-way ANOVA to identify the differences in bacterial taxa among all groups, or using student’s *t* test between two groups. The spearman correlation analysis was applied to evaluate the relationship of microbial species with the measured parameters. Correlations were considered significantly different at *P* ≤ 0.05. The analysis procedures of cecal microbiota were processed on the free online platform of Majorbio Cloud Platform (Majorbio Bio-pharm Technology Co., Ltd., Shanghai, China).

### Statistical Analysis

Cage was taken as experimental unit, and the homogeneity of variances of the data were tested before further analysis. One-way ANOVA followed by Duncan’s multiple range test were used to test the significant mean differences among groups. Polynomial orthogonal contrasts were applied to determine linearly and quadratically responses of measured parameters to the dietary FA concentration. All data analysis was performed in SPSS statistical program (SPSS25, IM Corp., Armonk, NY, United States). A probability of *P* < 0.05 was considered significant.

## Results

### Growth Performance

The initial BW, final BW, ADG, ADFI, and F/G of experimental ducks were shown in Table 2. The birds in Group 4 and 5 exhibited significantly higher final BW (*P* = 0.02) and ADG (*P* = 0.01) than the birds in Group 1. Group 3 showed the same trend, but the differences with Group 1 were not statistically significant. The F/G ratios in Group 3, 4, and 5 were decreased compared to Group 1 without statistical significance (*P* = 0.05). Significant linear (*P* < 0.05) and quadratic relationships (*P* < 0.05) were observed between final BW, ADG and F/G, and dietary FA level.

**TABLE 2:** Effect of different dietary FA supplementation on growth performance of experimental ducks^1^.

**Item**	**Treatments^2^**					**SEM**	***P* value**		
	**Group 1**	**Group 2**	**Group 3**	**Group 4**	**Group 5**		**ANOVA**	**Linear**	**Quadratic**
Initial BW, g	892.67	891.00	892.67	894.33	893.00	8.13	0.98	0.76	0.91
Final BW, g	1,553.33^bc^	1,542.33^c^	1,599.33^ab^	1,615.00^a^	1,609.67^a^	49.65	0.02	0.01	<0.01
ADG, g	22.78^c^	22.46^bc^	24.37^ab^	24.85^a^	24.71^a^	1.65	0.01	<0.01	<0.01
ADFI, g	149.00	150.98	150.97	152.15	151.72	6.98	0.96	0.56	0.74
F/G	6.57^ab^	6.73^a^	6.21^b^	6.13^b^	6.15^b^	0.45	0.05	0.03	0.04

*BW, body weight; ADG, average daily weight gain; ADFI, average daily feed intake; F/G, feed to gain ratio. ^*a to c*^Within a row, mean values bearing different superscripts differ significantly (*P* < 0.05). ^1^Data is the mean of six replicates per treatment. ^2^Group 1 (control), Group 2 (dietary FA supplementation at 100 mg/kg feed), Group 3 (dietary FA supplementation at 200 mg/kg feed), Group 4 (dietary FA supplementation at 400 mg/kg feed), and Group 5 (dietary FA supplementation at 800 mg/kg feed).*

### Serum Cytokines Profile: Oxidant and Antioxidant Status

The serum oxidant and antioxidant statuses were listed in Table 3. Serum levels of MDA (*P* = 0.52) and SOD activities (*P* = 0.22) among groups were similar, but significant differences were noticed in GSH levels (*P* = 0.01) and GSH-Px (*P* < 0.01) activities among groups. Serum levels of GSH in all groups except Group 4, and GSH-Px activities in all groups except Group 3 were significantly higher, compared to those in Group 1. Moreover, as the diet FA increased, the activities of GSH-Px increased linearly (*P* < 0.01) and quadratically (*P* < 0.01).

**TABLE 3:** Effect of different dietary FA supplementation on antioxidative parameters of experimental ducks^1^.

**Item**	**Treatments^2^**					**SEM**	***P* value**		
	**Group 1**	**Group 2**	**Group 3**	**Group 4**	**Group 5**		**ANOVA**	**Linear**	**Quadratic**
MDA, nmol/mL	3.19	2.55	2.49	2.81	2.43	0.83	0.52	0.32	0.55
GSH, μmol/L	76.64^c^	118.89^ab^	140.64^a^	93.14^bc^	137.48^a^	36.14	0.01	0.14	0.34
SOD, U/mL	154.85	151.26	137.25	151.55	152.65	14.36	0.22	0.78	0.49
GSH-Px, U/mL	580.74^c^	692.30^ab^	644.77^bc^	719.54^ab^	776.92^a^	99.97	<0.01	<0.01	<0.01

*^*a to c*^Within a row, mean values bearing different superscripts differ significantly (*P* < 0.05). MDA, malonaldehyde; GSH, glutathione; SOD, superoxide dismutase; GSH-Px, glutathione peroxidase. ^1^Data is the mean of six birds per treatment. ^2^Group 1 (control), Group 2 (dietary FA supplementation at 100 mg/kg feed), Group 3 (dietary FA supplementation at 200 mg/kg feed), Group 4 (dietary FA supplementation at 400 mg/kg feed), and Group 5 (dietary FA supplementation at 800 mg/kg feed).*

### Serum Cytokine Profile: Immune Status

As demonstrated in Table 4, there were no significant differences in serum levels of IgG (*P* = 0.28) and IL-1β (*P* = 0.51) among groups. However, Group 3, 4, and 5 showed significant lower levels of IL-6 (*P* = 0.04) and IL-2 (*P* = 0.02) comparing to Group 1. Negative linear (*P* < 0.05) and quadratic (*P* < 0.05) relationships were found between dietary FA concentration and the IL-6, as well as the IL-2 levels.

**TABLE 4:** Effect of different dietary FA supplementation on immunological parameters of experimental ducks^1^.

**Item**	**Treatments^2^**					**SEM**	***P* value**		
	**Group 1**	**Group 2**	**Group 3**	**Group 4**	**Group 5**		**ANOVA**	**Linear**	**Quadratic**
IgG, mg/mL	20.26	28.52	20.71	21.87	19.75	7.81	0.28	0.38	0.65
IL-1β, ng/mL	214.11	195.49	200.81	219.54	202.87	26.24	0.51	1.00	0.93
IL-6, ng/L	104.30^a^	94.31^ab^	78.20^b^	74.35^b^	78.98^b^	20.69	0.04	0.03	0.01
IL-2, ng/L	362.74^a^	242.06^ab^	223.16^b^	197.01^b^	139.50^b^	112.94	0.02	<0.01	<0.01

*^*a to b*^ Within a row, mean values bearing different superscripts differ significantly (*P* < 0.05). IgG, immunoglobulin G; IL-1β, interlukin-1β; IL-6, interlukin-6; IL-2, interleukin-2. ^1^Data is the mean of six birds per treatment. ^2^Group 1 (control), Group 2 (dietary FA supplementation at 100 mg/kg feed), Group 3 (dietary FA supplementation at 200 mg/kg feed), Group 4 (dietary FA supplementation at 400 mg/kg feed), and Group 5 (dietary FA supplementation at 800 mg/kg feed).*

### Intestinal Morphology

**FIGURE 1:**
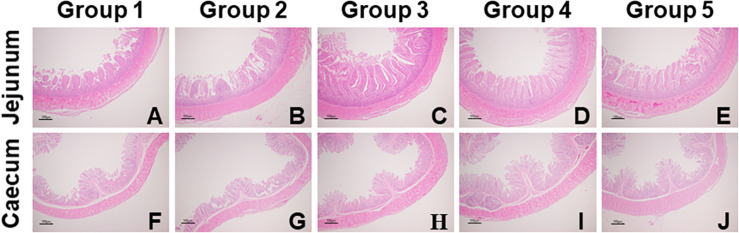
Representative images of jejunal **(A–E)** and cecal **(F–J)** morphology of experimental ducks in different groups (40 × magnification). Intestinal issue samples were processed with hematoxylin and eosin stain.

As shown in Table 5, compared to Group 1, Group 4 and 5 showed significantly decreased crypt depth in the jejunum (*P* = 0.01); Group 3, 4, and 5 showed significantly increased VCR in the jejunum (*P* = 0.02); and Group 4 showed significantly decreased crypt depth in the caecum (*P* = 0.04). Significant linear (*P* < 0.01) and quadratic (*P* < 0.01) relationships were noticed between dietary FA and the crypt depth (negative) and VCR (positive) in the jejunum; and significant quadratic but no linear relationship were found between the dietary FA and the crypt depth in the cecum (*P* < 0.05). In the representative images of jejunum and cecum tissues, it was noticeable that the intestinal mucosae in FA treated groups were more intact than those in Group 1 (Figure 1).

**TABLE 5:** Effect of different dietary FA supplementation on intestinal morphology of experimental ducks^1^.

**Item**	**Treatments^2^**					**SEM**	***P* value**		
	**Group 1**	**Group 2**	**Group 3**	**Group 4**	**Group 5**		**ANOVA**	**Linear**	**Quadratic**
**Duodenum**									
VH, μm	429.59	417.97	328.82	404.75	413.75	77.62	0.16	0.59	0.71
CD, μm	100.15	111.71	90.10	103.49	107.56	15.53	0.14	0.30	0.49
VCR	4.39	3.76	3.65	3.96	3.88	0.79	0.56	0.43	0.33
IWT, μm	193.89	212.70	195.87	227.04	205.50	42.56	0.69	0.31	0.58
MT, μm	601.66	632.23	531.90	595.67	626.65	84.25	0.15	0.91	0.40
**Jejunum**									
VH, μm	343.11	363.83	357.62	350.50	350.54	54.25	0.98	0.66	0.91
CD, μm	101.44^a^	102.36^a^	89.10^ab^	86.91^b^	81.69^b^	13.38	0.01	<0.01	<0.01
VCR	3.36^c^	3.58^bc^	4.00^ab^	4.06^ab^	4.35^a^	0.59	0.02	<0.01	<0.01
IWT, μm	198.48	199.71	182.19	216.91	197.60	47.26	0.83	0.97	0.91
MT, μm	494.59	517.67	486.43	492.01	499.14	73.48	0.97	0.45	0.64
**Ileum**									
VH, μm	310.43	366.56	380.75	306.50	326.86	65.81	0.16	0.61	0.61
CD, μm	82.33	90.13	97.94	87.00	89.42	11.00	0.17	0.45	0.16
VCR	3.80	4.03	3.91	3.51	4.10	0.60	0.50	0.93	0.90
IWT, μm	183.44	227.78	224.17	196.97	195.51	38.29	0.19	0.90	0.14
MT, μm	444.07	536.80	524.59	454.07	497.22	91.48	0.31	0.85	0.54
**Caecum**									
VH, μm	191.94	189.09	197.99	182.21	173.82	28.93	0.68	0.26	0.38
CD, μm	53.77^a^	50.38^ab^	50.34^ab^	47.16^b^	56.48^a^	5.77	0.04	0.77	0.03
VCR	3.59	3.75	3.94	3.94	3.08	0.66	0.12	0.34	0.04
IWT, μm	165.01	151.48	165.03	154.10	146.35	25.72	0.67	0.30	0.57
MT, μm	237.08	251.93	272.55	243.90	237.15	34.03	0.36	0.86	0.21

*VH, villus height; CD, crypt depth; VCR, ratio of villus height to crypt depth; IWT, intestinal wall thickness; MT, mucosa thickness. ^*a to c*^Within a row, mean values bearing different superscripts differ significantly (*P* < 0.05). ^1^Data is the mean of six birds per treatment. ^2^Group 1 (control), Group 2 (dietary FA supplementation at 100 mg/kg feed), Group 3 (dietary FA supplementation at 200 mg/kg feed), Group 4 (dietary FA supplementation at 400 mg/kg feed), and Group 5 (dietary FA supplementation at 800 mg/kg feed).*

### Modulation of Gut Microbiota

**FIGURE 2:**
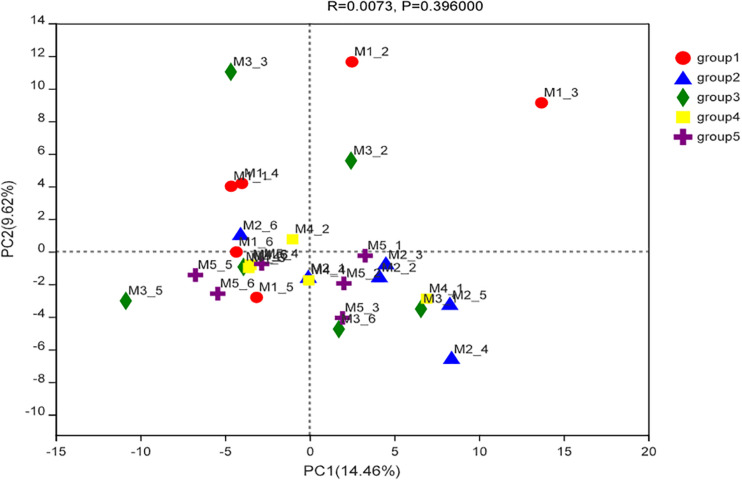
Principal component analysis ordinated plots of cecal microbial composition at the genera level of ducks in different groups.

The compositions of cecal microbiota in all groups were analyzed by 16S rRNA sequencing. According to the α diversity result (Table 6), the cecal microbial abundances or diversities at the genera level were similar among groups (*P* > 0.05). PCA and ANOSIM further revealed no clear clustering patterns for cecal microbiota at genera level among groups (*P* = 0.40), as showed in Figure 2.

**TABLE 6:** The parameters of α diversity of cecal microbiota in different groups^1^.

**Parameter**	**Treatments^2^**					**SEM**	***P* value**
	**Group 1**	**Group 2**	**Group 3**	**Group 4**	**Group 5**		
Shannon	3.28	3.27	3.22	3.18	3.31	0.28	0.95
Simpson	0.085	0.090	0.087	0.098	0.081	0.032	0.94
ACE	142.49	146.99	143.85	143.44	144.66	13.74	0.99
Chao	140.03	149.85	149.57	147.77	145.21	18.81	0.91

*^1^Data is the mean of six birds per treatment. ^2^Group 1 (control), Group 2 (dietary FA supplementation at 100 mg/kg feed), Group 3 (dietary FA supplementation at 200 mg/kg feed), Group 4 (dietary FA supplementation at 400 mg/kg feed), and Group 5 (dietary FA supplementation at 800 mg/kg feed).*

**FIGURE 3:**
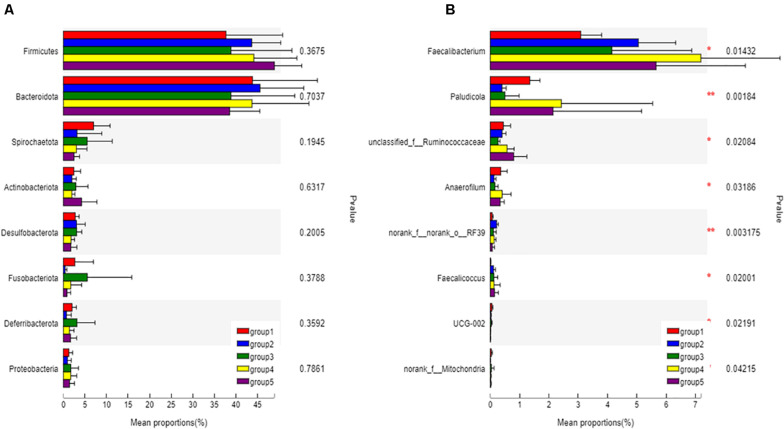
Classification and differences in cecal microbial composition of ducks in different groups. The eight most abundant bacteria at the phyla level **(A)** and bacteria with significant difference at genus level **(B)** among five groups were showed. Relative abundance of altered cecal microbiota levels were tested under ANOVA and Tukey *post hoc* analysis. Significant correlations were marked by *0.01 < *P* < 0.05, **0.001 < *P* ≤ 0.01.

**FIGURE 4:**
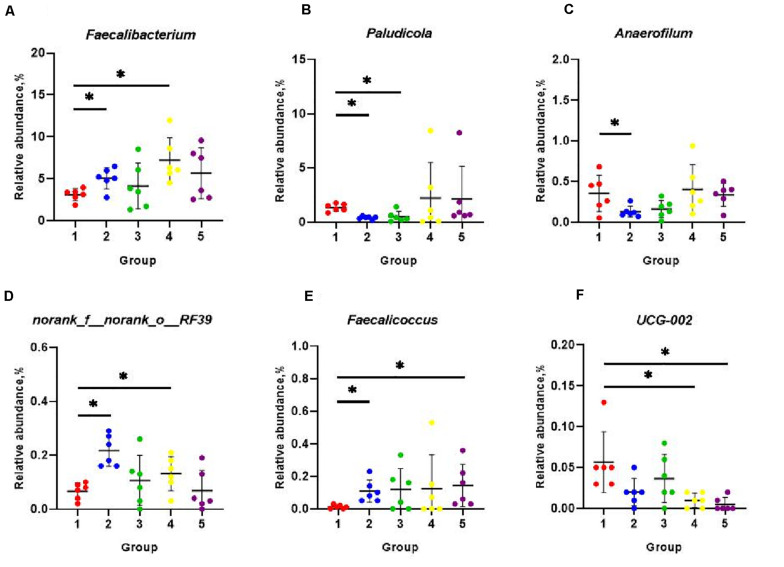
Classification and differences in cecal microbial composition at the genera level among five groups. The relative abundance of microbial genera that significantly altered included *Faecalibacterium* **(A)**, *Paludicola* **(B)**, *Anaerofilum* **(C)**, norank_f_norank_o_RF39 **(D)**, *Faecalicoccus* **(E)**, and UCG-002 **(F)**. Data were shown as means ± SD (*n* = 6), *P < 0.05 compared with Group 1.

The eight most predominant microbial phyla among groups were *Firmicutes* (43.3–48.7%), *Bacteroidetes* (40.5–47.8%), *Spirochaetes* (1.0–6.5%), *Actinobacteriota* (1.9–3.2%), *Desulfobacterota* (1.0–3.0%), *Fusobacteriota* (0.4–5.6%), *Deferribacterota* (0.1–3.7%), and *Proteobacteria* (0.9–1.7%) (Figure 3A). No significant differences in mean proportion were found in microbial communities at the phyla level among groups (*P* > 0.05). But at genera level, a total of eight microbial genera were found significantly different among (*P* < 0.05) groups (Figure 3B). Pair-wise comparisons with Student’s *T* test for the differences in microbial composition at genus level were further conducted between Group 1 and FA treated groups. Significant differences were found in proportions of *Faecalibacterium*, *Paludicola*, *Anaerofilum*, RF39, and *Faecalicoccus* between Group 1 and 2 (*P* < 0.05) (Figures 4A–E), *Paludicola* between Group 1 and 3 (*P* < 0.05) (Figure 4B), *Faecalibacterium*, RF39, and UCG-002 between Group 1 and 4 (*P* < 0.05) (Figures 4A,D,F), and *Faecalicoccus* and UCG-002 between Group 1 and 5 (*P* < 0.05) (Figures 4E,F).

**FIGURE 5:**
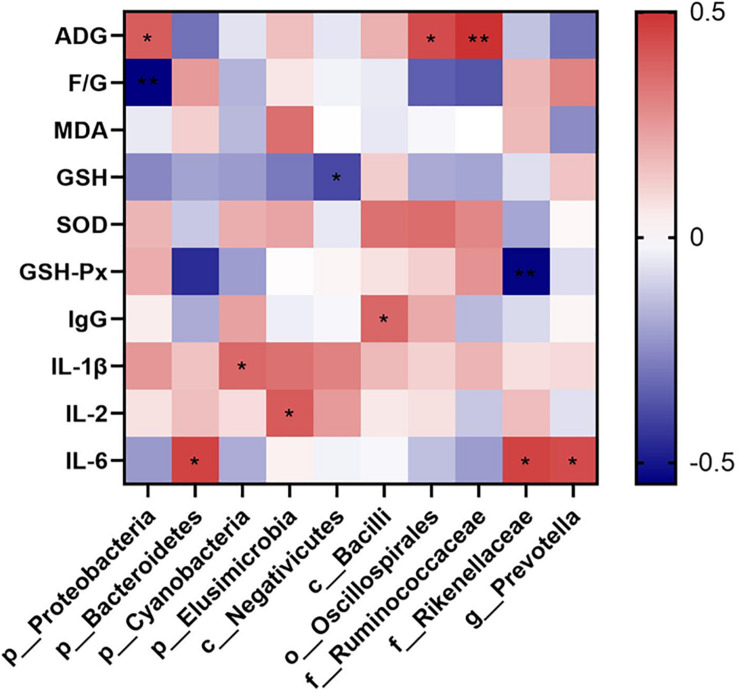
Spearman correlation heatmap of cecal microbiota and the measured parameters. The intensity of the colors demonstrated the degree of association (blue, negative correlation; red, positive correlation). Significant correlations marked by *0.01 < *P* ≤ 0.05, **0.001 < *P* ≤ 0.01. ADG, average daily weight gain; F/G, ratio of feed intake to weight gain; MDA, malonaldehyde; GSH, glutathione; SOD, superoxide dismutase; GSH-Px, glutathione peroxidase; IgG, immunoglobulin G; IL-1β, interlukin-1β; IL-6, interlukin-6; IL-2, interleukin-2.

The relationships between the cecal microbiota and the parameters relating to the growth performance, levels of cytokines representing the oxidative and inflammatory statuses were examined *via* Spearman’s correlation analysis. Data were showed in Figure 5. ADG was positively associated with the phylum *Proteobacteria* (*P* = 0.035), order *Oscillospirales* (*P* = 0.018), and family *Ruminococcaceae* (*P* = 0.006); and F/G was negatively associated with the phylum *Proteobacteria* (*P* = 0.002). Additionally, GSH level was negatively associated with the class *Negativicutes* (*P* = 0.036); and GSH-Px activity was negatively associated with the phylum *Bacteroidetes* (*P* = 0.013) and family *Rikenellaceae* (*P* = 0.003). Finally, IgG level was positively associated with the class *Bacilli* (*P* = 0.049); IL-1β level was positively associated with the phylum *Cyanobacteria* (*P* = 0.050); IL-2 level was positively associated with the phylum *Elusimicrobia* (*P* = 0.032); and IL-6 level was positively associated with phylum *Bacteroidetes* (*P* = 0.012), family *Rikenellaceae* (*P* = 0.013), and genus *Prevotella* (*P* = 0.017).

## Discussion

Stresses were the main factors that deteriorated the profits in poultry industry as the intensive system developing (Vizzier Thaxton et al., 2016). FA was reported to be able to ameliorate the oxidative stress by capturing reactive oxygen species (ROS) with its structure characteristics (Maurya and Devasagayam, 2010), and triggering the productions of the antioxidant enzymes through Nrf2-Keap1-ARE signaling pathway (Krajka-Kuźniak et al., 2015). Additionally, it was reported that FA could mediated the inflammation through IKK/NF-κB signaling pathway (Lampiasi and Montana, 2016). Some researchers utilized FA as feed additive in livestock farming and proved its beneficial effects. Wang et al. (2020) found that 0.05 and 0.45% FA supplemented in the diet improved the lipid profile and antioxidant capacity of the weaning piglets. González-Ríos et al. (2016) reported that steers fed diet with 6 mg/kg FA supplementation in the last 30 days of the finishing phase acquired better meat quality, and delayed lipid oxidation during storage. However, few papers were published relating to the effects of FA on poultry. In the present study, significant increase in ADG, and decrease in F/G ratio in ducks that fed diets supplemented with 200–800 mg/kg FA were detected during the growing stage (28–56 days of age). And the trends of changes were linear and quadratic related to the FA supplement rate. These results suggested promotional effects of FA on the growth performance of the ducks. As the writer’s knowledge, this study is the first time using pure FA as feed additive in poultry experiment. Previous researchers tried diets supplemented with propolis, sorghum or other materials, of which FA was considered as the main substances, with birds. Positive results were found with propolis that they improved the feed efficiency and ameliorated the effects of stresses on the weight gain (Seven, 2008; Hassan and Abdulla, 2011; Abdel-Rahman, 2013), which corroborated with our finding. However, birds offered sorghum-based diets showed decreased body weight gains (Truong et al., 2016). The variations in the effects of FA enriched materials on bird’s growth performance could partly be explained by the condensed tannins, other than the FA in the sorghum, negatively influenced energy utilization of starch, so that the weight gain of the birds decreased. It was also reported that FA in conjugated forms in plants might form complexes with debranched starch which caused the resistance to digestion (Hung et al., 2012).

In the present study, serum parameters related to the avian antioxidative status were investigated. Oxidative stress was mainly caused by the excessive accumulations of the ROS. In the organism, redox balance was modulated by the antioxidant defense system, consisting of enzymatic components such as SOD and GSH-Px, and non-enzymatic components such as GSH (Surai et al., 2019). Once the accumulation of ROS overpassed the scavenging capability of antioxidant defense system, the redox balance would break and the oxidative stress occurred (Nisar et al., 2013). MDA was formed by ROS degrading the polyunsaturated lipids in the organism, and was considered as a common biomarker for oxidative stress (Fu et al., 2013). The present study revealed the antioxidant effects of dietary FA, as it significantly improved the serum GSH levels and GSH-Px activities. Significant linear and quadratic relationships were noticed between the activities of GSH-Px and the rate of dietary FA. Similar results were found in multiple studies. The hepatic GSH contents and activities of SOD, catalase (CAT) and GSH-Px in methotrexate (MTX) induced rats were significantly alleviated after 15 days of oral supplements of 25 or 50 mg/kg FA (Mahmoud et al., 2020). It was also published that diets with 0.45% FA supplementation decreased the MDA contents, and increased the activities of CAT, total SOD and GSH-Px in the serums and the livers of the weaning pigs (Wang et al., 2020). Moreover, Wang et al. (2019) reported that 3-m-old lambs fed diet supplemented with 80 mg/kg FA showed higher plasma levels of GSH-Px, CAT and lower levels of MDA compared to the lambs fed basal diet.

Immunological stress was another notorious factor that deteriorated the animal performance (Lochmiller and Deerenberg, 2000). It usually occurred when animals were challenged by infectious agents, such as pathogenic organisms or environmental insults (Song et al., 2014). When immunological stress happened, both innate and adaptive systems in birds were triggered. Avian heterophils with strong phagocytic activities were firstly activated (Genovese et al., 2013). Later the basophils (Maxwell and Robertson, 1995), eosinophils (Maxwell, 1987), and dendritic cells (Ma S. et al., 2019) were involved as early modulators of inflammation and/or antigen presenters who initiated the adaptive immune responses. Lymphocyte cells in avian adaptive immune system were able to produce a diversity of antibodies, such as immunoglobulin M (IgM), IgG, and IgA, which served as the first line to fight against the intruders (Berghof et al., 2018). Simultaneously, the production of cytokines, such as IL-1β, IL-6, IL-2, were increased as chemical messengers that affected the proliferation, differentiation and activity of immune cells (Kaiser et al., 2005). In the present study, the addition of FA in diets significantly decreased the serum levels of IL-6 and IL-2, which outlined that the immunological status in the organism was ameliorated. Additionally, significant linear and quadratic relationships were noticed between the concentrations of dietary FA and the levels of the two cytokines. These results were partially consistent with previous findings in rats (Sadar et al., 2016; Mahmoud et al., 2020) and *in vitro* cell model (Cao et al., 2015), as FA attenuated not only the level of IL-6 but also IL-1β in the pathogenic circumstances.

Gastrointestinal tract (GIT) was essential for the growth of animals, and its integrity was a key factor for preventing pathogenic microorganism invasion and utilizing the nutrients (Choct, 2009). Villus height reflected the intestinal absorptive capacities of the nutrients and the crypt depth represented the colonization rate and the maturities of the crypt cells (Feng et al., 2017). The ratio of these two parameters was regarded as a general indicator for the intestinal function. Additionally, intestinal wall and mucosa were the foundations to GIT’s barrier function, of which the thickness was extremely critical. In our study, 200–800 mg/kg dietary FA supplementation linearly and quadratically decreased the crypt depth and increased the VCR of the jejunum, and 100–400 mg/kg dietary FA quadratically decreased the crypt depth of the caecum, demonstrating enhancing effects of dietary FA on the intestinal morphology in the ducks. This result was supported by previous research that 50 mg FA/kg body weight attenuated the effects of heat stress on rats’ intestinal permeability and microvilli structure (He et al., 2016). It was further reported that the beneficial effects of FA on the intestinal morphology and barrier function were possibly contributed to the activation of Nrf2/HO-1 signaling pathway, which was associated to the process of ROS elimination as well (He et al., 2018).

Microorganism inhabiting in the GIT were involved in the digestion of food, breakdown of toxins, stimulation of immune system, exclusion of pathogens and endocrine activities. The interaction between the microorganism and GIT influenced the stability of microbial communities, the animal’s health and the feed efficiency (Apajalahti et al., 2004). Cecum was the main location for the fermentation and digestion of complex substrates, so that it possesses the longest feed retention time and most diverse microorganism (Borda-Molina et al., 2018). In order to understand the effects of FA on the gut microbial environment, the chyme in the cecum were chosen in the present study for microbial community analysis. FA was reported to modulate gut microbiota composition in transverse aortic construction mice (Liu et al., 2019) and diabetic mice model (Ma y. et al., 2019; Huang et al., 2020). In present study, the differences of richness and diversity of cecal microbial community among groups were not statistically significant. The possible explanation to the inconsistencies might be the constructional and functional differences of the GIT between birds and mammals, as well as the sensitivities of the microbiome to FA in the diverse animal species. Previous papers illustrated that cecal bacterial microbiome of birds were usually dominated by members of the phyla *Bacteroidetes* and *Firmicutes*, followed by lower abundance of *Proteobacteria*, *Actinobacteria* and others (Costa et al., 2017; She et al., 2018; Xia et al., 2019). It was consistence with the cecal microbial construction in the present study. All groups in our study showed similar patterns of cecal microbial compositions at phyla level, but the changes in relative abundance of some bacteria at genera level were detected. Pair-wise comparison between the FA treated and untreated groups further suggested that different rates of dietary FA could alter the microbial compositions in the cecum. It was well documented that the intestinal microbiome closely interacted with the host through the exchange of nutrients, modulation of gut morphology and physiology, and maintenance of immune and redox homeostasis (Oakley et al., 2014; Pan and Yu, 2014; He et al., 2019)014). In the present study, the relative abundances of *Faecalibacterium* and *Faecalicoccus* were significantly increased with 400 and 800 mg/kg FA supplement, respectively. And the UCG-002 level was decreased in these two groups. Correspondingly, the ADGs were significantly higher in Group 4 (400 mg/kg FA) and 5 (800 mg/kg FA) compared to Group 1. Literatures indicated that species in *Faecalibacterium* were beneficial that improved the epithelial health and promoted the metabolite productions, especially butyrate and other SCFAs (Gangadoo et al., 2018), and *Faecalicoccus* belonged to family *Erysipelotrichaceae*, which was reported to be a major butyrate producer and considered more important in poultry cecum than those in human colon (Zhou et al., 2021). UCG-002 belongs to the family *Oscillospiraceae*, which was reported to be negatively related to the body weight gain of geese fed fermented feed (Yan et al., 2019). These studies were consistent with what we found, showing that the trends of changes in abundance of bacteria taxa mentioned above were correlative to the improvement in growth performance. *Anaerofilum* were published to be positively associated with the body weight gain in broilers fed dietary vitamins (Luo et al., 2013). But in the present study, we found inconsistent data regarding to the level of *Anaerofilum* and the body weight gains of the birds. *Paludicola* were a novel genus in the family *Ruminococcaceae*, whose function in the gut was unknown yet (Li et al., 2017).

The Spearman correlation analysis disclosed that phylum *Proteobacteria*, order *Oscillospirales*, and family *Ruminococcaceae* were associated with the ADG, and phylum *Proteobacteria* were associated with the F/G ratio, which demonstrated the strong influences of such bacteria on the growth performance in ducks. Similar results were reported previously regarding to those bacteria. Though phylum *Proteobacteria* included several pathogens, they could degrade the nutrients in the diet and provide suitable environmental support for symbiotic bacteria, and eventually promoted the growth of layers (Li et al., 2020). Shi et al. (2019) stated that species in order *Oscillospirales*, such as *Oscillospira*, were butyrate producers and negatively related to the F/G ratio in broilers under heat stress. Additionally, major members in family *Ruminococcaceae* were short-chain fatty acids producing bacteria, and they were outstanding for improving feed efficiency and enhancing health for the host (Wong et al., 2006). In the present study, class *Negativicutes* was found negatively associated with the GSH levels, and phylum *Bacteroidetes* and family *Rikenellaceae* were negatively associated with the GSH-Px activities. These species might be possible positive symbols to the levels of oxidative stress of the host. It was consistent with previous studies that the relative abundances of class *Negativicutes* were reduced in ducks with oxidative stress caused by heat (He et al., 2019), phylum *Bacteroidetes* were positively related to oxidative stress in rats fed high-fat diets (Ortega-Hernández et al., 2020), and relative abundance of family *Rikenellaceae* decreased after polysaccharides from pollen of Chinese wolfberry, which attenuated the oxidative stress in cyclophosphamide-treated mice (Zhao et al., 2020). Moreover, we found that phylum *Bacteroidetes*, family *Rikenllaceae* and genus *Prevotella* were positively associated with IL-6 levels; and class *Bacilli*, phylum *Cyanobacteria*, and phylum *Elusimicrobia* were positively associated with IgG, IL-1β, and IL-2 levels respectively. These findings potentially indicated the positive relationships of the relative abundances of these markers to the levels of immune stress of the host. Besides the roles of representative to the oxidative stress, phylum *Bacteroidetes* were reported to be implicated in immune regulations including activations of inflammation and autoimmune diseases (Gibiino et al., 2018), and family *Rikenellaceae* were proved to be positive associated with the arise of immune response induced by *Pleurotus eryngii* polysaccharide in mice (Ma et al., 2017). *In vitro* study using human monocyte-derived dendritic cells proved that genus *Prevotella* exhibited capacities to induce inflammatory mediators like IL-6 (Horewicz et al., 2013). Additionally, members of family *bacilli*, including the Gram-negative *bacilli* were among the most common causative agents of infectious diseases, and triggers of the inflammation (Dandachi et al., 2019). Phylum *Cyanobacteria* were reported to be immune suppressing (Gemma et al., 2016), but some members in *Cyanobacteria*, for example *Microcystis aeruginosa*, whose lipopolysaccharide could trigger human immune response (Moosová et al., 2019). Moreover, phylum *Elusimicrobia* were proved to be positively correlated to multiple proinflammatory cytokines and promoted the occurrences of immunological stress in colitis rats (Tao et al., 2017), which was in line with our finding.

Even so, massive works were still required to clarify the mechanism of the influences of FA on ducks. The obtained alternations of microbial species associated with the dietary FA should be carefully validated. The molecular pathway associated with the amelioration of oxidative and immunological stress by FA was needed to be explored. And the participation of microbiota in the stress modulation still required to be discussed.

## Conclusion

In conclusion, dietary supplementation of FA altered the intestinal microbiota at the genera level, which associated with the beneficial effects on the growth performance, anti-oxidant and anti-inflammatory capabilities, intestinal morphology, and epithelia barrier functions in *Linwu* ducks at the growing stage. Quantity effects were obvious as the significant linear and quadratic relationships were noticed between diet FA levels and the measured parameters. Considering the cost and the efficacy of dietary FA as feed additives, we concluded that 400 mg/kg was the suggested supplementation rate of FA in diets for *Linwu* ducks during growing stage for the future use as feed additive.

## Data Availability Statement

The datasets presented in this study can be found in online repositories. The names of the repository/repositories and accession number(s) can be found below: https://www.ncbi.nlm.nih.gov/, PRJNA723283.

## Ethics Statement

The animal study was reviewed and approved by Hunan Agricultural University Institutional Animal Care and Use Committee.

## Author Contributions

QD and XGH conceived of and designed the experiments. YL, XH, CL, and GJ performed the sampling. XH performed the serum index measurement. XZ conducted the tissue section. QL performed the 16S rRNA sequencing experiment. YLL, QL, SL, and LH analyzed the data. YL wrote the manuscript. QL contributed to refining the text. All authors contributed to the article and approved the submitted version.

## Conflict of Interest

SL was employed by the company Shandong Lonct Enzymes Co., Ltd. YLL was employed by the company Hunan Perfly Biotech Co., Ltd. The remaining authors declare that the research was conducted in the absence of any commercial or financial relationships that could be construed as a potential conflict of interest.
